# Effect of semaglutide on arrhythmic, major cardiovascular, and microvascular outcomes in patients with type 2 diabetes: a systematic review and meta-analysis

**DOI:** 10.3389/fendo.2025.1554795

**Published:** 2025-08-26

**Authors:** Rui Wu, Bo Xing, Yuting Huang, Zijun Zhou, Boxuan Sun, Liming Yu, Huishan Wang

**Affiliations:** ^1^ School of Life Sciences and Biopharmaceuticals, Shenyang Pharmaceutical University, Shenyang, Liaoning, China; ^2^ State Key Laboratory of Frigid Zone Cardiovascular Disease, Department of Cardiovascular Surgery, General Hospital of Northern Theater Command, Shenyang, Liaoning, China

**Keywords:** semaglutide, arrhythmic, cardiovascular, microvascular, type 2 diabetes, meta-analysis

## Abstract

**Background:**

Semaglutide, a glucagon-like peptide-1 (GLP-1) receptor agonist, has shown promise in managing hyperglycemia and reducing cardiovascular (CV) outcomes. However, its effects on arrhythmic, major CV, and microvascular outcomes remain uncertain. This systematic review and meta-analysis aimed to evaluate these outcomes in patients with type 2 diabetes (T2D) treated with semaglutide.

**Methods:**

We searched the PubMed, Embase, and Cochrane databases for eligible randomized controlled trials (RCTs) reported up to November 2024. We performed a meta-analysis via a random-effects model to estimate overall relative risks (RRs) with 95% confidence intervals (CIs) for arrhythmic, major CV, and microvascular outcomes. We conducted subgroup analyses on the basis of different administration types, treatment comparisons, and treatment durations. Additionally, we performed a meta-regression for retinopathy complications on the basis of baseline patient characteristics.

**Results:**

This meta-analysis included 30 RCTs encompassing 32490 patients with T2D. Compared with the controls, semaglutide significantly reduced the incidence of atrial fibrillation (AF) (RR 0.73, 95% CI 0.54 to 0.98), complete atrioventricular (AV) block (RR 0.22, 95% CI 0.06 to 0.80), death from CV causes (RR 0.76, 95% CI 0.58 to 0.98), and revascularization (RR 0.68, 95% CI 0.52 to 0.88). Subgroup analyses revealed that semaglutide (long-term treatment) reduced the risk of AF, supraventricular tachycardia, and complete AV block. Meta-regression analysis revealed that the heterogeneity of retinopathy complications was not associated with baseline patient characteristics.

**Conclusion:**

Semaglutide reduces the risk of AF, complete AV block, death from CV causes, and revascularization in patients with T2D, with long-term treatment showing greater benefits for arrhythmic outcomes.

**Systematic review registration:**

https://www.crd.york.ac.uk/prospero/, identifier CRD42024618146.

## 1 Introduction

Type 2 diabetes (T2D) is a complex and chronic metabolic disorder characterized by insulin resistance, progressive beta-cell dysfunction, and persistent hyperglycemia. The prevalence of T2D is increasing worldwide, and T2D poses a significant public health burden. Patients with T2D are at heightened risk for cardiovascular (CV) disease, which remains the leading cause of morbidity and mortality in this population (1, 2). Among the various arrhythmic outcomes, atrial fibrillation (AF), ventricular tachycardia, and atrioventricular (AV) block are common and further increase the risk of stroke, heart failure (HF), and death. Furthermore, major CV outcomes, including nonfatal myocardial infarction, nonfatal stroke, and revascularization, contribute to the high burden in patients with T2D. Microvascular outcomes, such as diabetic retinopathy and nephropathy, also present significant challenges, leading to impaired quality of life and increased healthcare costs.

Over the past decade, glucagon-like peptide-1 (GLP-1) receptor agonists have emerged as a promising class of drugs for the management of T2D (3). In addition to their glucose-lowering effects, GLP-1 receptor agonists, including semaglutide, have demonstrated pleiotropic benefits in reducing body weight, improving lipid profiles, and lowering blood pressure. Semaglutide, a long-acting GLP-1 receptor agonist, has gained attention for its potential cardioprotective properties (4). Clinical trials have reported its efficacy in reducing major CV outcomes, particularly death from CV causes (5), and improving glycemic control in patients with T2D (6). There is also a review that details all the research conducted on semaglutide, emphasizing its effects on CV outcomes in patients with T2D (7). Notably, one study has shown that semaglutide reduces the risk of AF (8), but its effect on other arrhythmic and microvascular outcomes remains uncertain.

To address these gaps, this systematic review and meta-analysis represents the most up-to-date and comprehensive evaluation of the effects of semaglutide on arrhythmic, major CV, and microvascular outcomes in patients with T2D. Unlike previous analyses, our study places a particular emphasis on arrhythmic outcomes, providing a more detailed understanding of the effects of semaglutide on various types of arrhythmias. This work not only highlights the benefits and limitations of semaglutide but also underscores its potential role in managing CV outcomes comprehensively while identifying areas for future research to refine its therapeutic applications.

## 2 Materials and methods

This meta-analysis was performed according to the Preferred Reporting Items for Systematic Reviews and Meta-Analyses (PRISMA) guidelines (9) and has been registered at PROSPERO (CRD42024618146).

### 2.1 Data sources and search strategy

We searched PubMed, Embase, and the Cochrane Library until 25 November 2024. The keywords searched were T2D, semaglutide, and randomized controlled trials (RCTs). The detailed search strategy can be found in **Supplementary Table 1**.

### 2.2 Inclusion and exclusion criteria

Eligible studies included the following: (1) the studies were randomized, controlled, and interventional; (2) the population of interest to the studies was patients with T2D; (3) the studies reported arrhythmic or major CV or microvascular outcomes; and (4) semaglutide in the intervention group and placebo or other drugs in the control group. In contrast, studies with unpublished results, duplications of prior publications, case reports, or conference abstracts were excluded.

### 2.3 Quality assessment

We used the Cochrane Collaboration’s risk of bias tool (10) to assess the risk of bias for individual trials. The tool states that bias can arise from 7 aspects, namely, selection bias, performance bias, detection bias, attrition bias, reporting bias and other bias. For each aspect, studies were categorized as high, unclear, or low risk of bias.

### 2.4 Data extraction

The following information was extracted from the included studies: first author, year, country, age, weight, body mass index (BMI), duration of diabetes, different administration types, treatment comparisons, and treatment durations. We are interested in three main areas: arrhythmic outcomes, major CV outcomes, and microvascular outcomes. The arrhythmic outcomes included AF, atrial flutter (AFL), ventricular tachycardia, supraventricular tachycardia, ventricular fibrillation, sinus node dysfunction, first degree AV block, second degree AV block, and complete AV block; major CV outcomes included nonfatal myocardial infarction, nonfatal stroke, death from CV causes, unstable angina pectoris, revascularization, and HF; and microvascular outcomes involved retinopathy complications, and new or worsening nephropathy.

### 2.5 Statistical analysis

Dichotomous outcomes are quantified by calculating relative risks (RRs) coupled with 95% confidence intervals (CIs). Statistical heterogeneity was measured via the chi-square test and *I* ^2^ statistic. Given the expected heterogeneity among the studies included in this meta-analysis, a random-effects model was employed to combine effect estimates. This approach is appropriate as it accounts for both within-study and between-study heterogeneity, allowing for more generalized conclusions that consider differences in study populations, interventions, and outcomes (11). Subgroup analyses were conducted to explore the effects of different administration types, treatment comparisons, and treatment durations on outcomes. For outcomes that included 10 or more studies, publication bias was assessed via Egger’s test, and regression analyses were performed on the basis of patient age, weight, BMI, and duration of diabetes to explore potential heterogeneity. Differences were considered statistically significant when *p* < 0.05. All the statistical calculations were performed via Stata software version 12.0 (StataCorp, College Station, TX, USA).

## 3 Results

### 3.1 Study selection and characteristics

**Figure 1:**
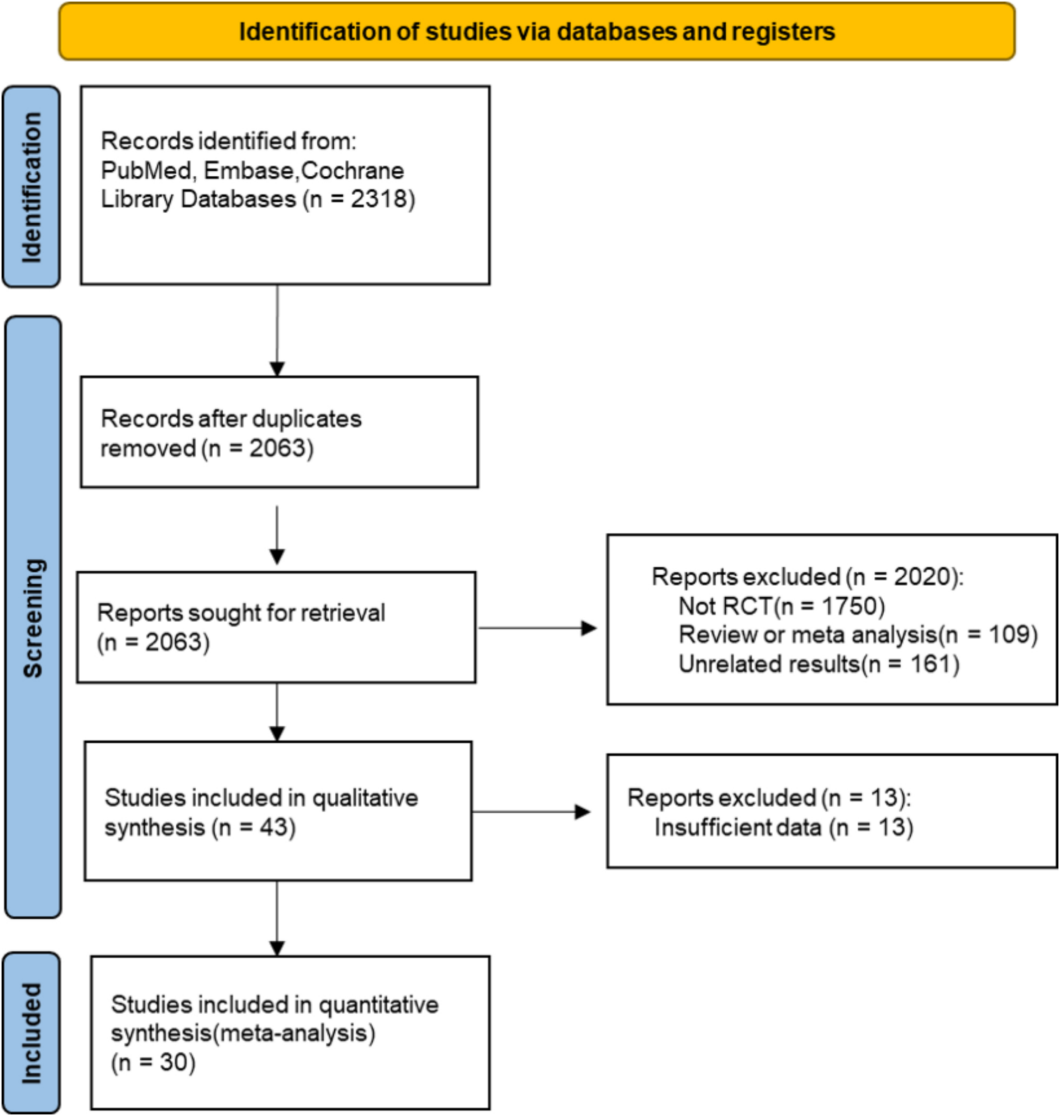
Flowchart of study selection.

From the comprehensive database search, 2318 articles were initially identified, and after the exclusion of duplicate studies, 2063 remained for further consideration. Next, 2020 articles were excluded for irrelevant reviews or non-RCTs. A thorough review of the full texts of the remaining 43 articles revealed that 13 articles were excluded because of insufficient data to support our research objectives. Specifically, these studies were excluded for the following reasons: (1) lack of reporting on relevant outcomes (e.g., absence of arrhythmic, major CV, or microvascular outcome data); (2) inadequate sample sizes (e.g., fewer than 50 participants); or (3) incomplete data reporting that hindered comprehensive analysis of the intervention’s effects. These exclusion criteria ensured that we ultimately included 30 articles (12–41) in the meta-analysis. The selection process for the study is shown in **Figure 1**, and the basic characteristics of the included studies are detailed in **Table 1**.

**Table 1:** Basic characteristics of the eligible studies.

Study	Acronym	NCT number	Age (yr)	Weight (kg)	BMI (kg/m^2^)	Durationof diabetes (yr)	Semaglutide intervention	Semaglutide dose (mg)	Frequency	Control intervention	Semaglutide group(N)	Control group(N)	Duration (w)
(12)	SUSTAIN 6	NCT01720446	64.6	92.1	–	13.9	Subcutaneous	0.5 and 1	once-weekly	Placebo	1648	1649	104
(13)	SUSTAIN 2	NCT01930188	55.1	89.5	32.5	6.6	Subcutaneous	0.5 and 1	once-weekly	Sitagliptin	818	407	56
(14)	SUSTAIN 4	NCT02128932	56.5	93.5	33.0	8.6	Subcutaneous	0.5 and 1	once-weekly	Insulin	722	360	30
(15)	SUSTAIN 1	NCT02054897	53.7	91.9	32.9	4.2	Subcutaneous	0.5 and 1	once-weekly	Placebo	258	129	30
(20)	SUSTAIN™	NCT02254291	58.3	69.3	25.4	8.0	Subcutaneous	0.5 and 1	once-weekly	Sitagliptin	205	103	30
(17)	SUSTAIN™	NCT02207374	58.5	71.5	26.4	8.8	Subcutaneous	0.5 and 1	once-weekly	Other oral antihyperglycemic drug	480	120	56
(16)	SUSTAIN 3	NCT01885208	56.6	95.8	33.8	9.2	Subcutaneous	1	once-weekly	Exenatide	404	405	56
(18)	SUSTAIN 7	NCT02648204	56.0	95.2	33.5	7.4	Subcutaneous	0.5 and 1	once-weekly	Dulaglutide	601	598	40
(19)	SUSTAIN 5	NCT02305381	58.8	91.7	32.2	13.3	Subcutaneous	0.5 and 1	once-weekly	Placebo	263	133	30
(27)	PIONEER 2	NCT02863328	58.0	91.6	32.8	7.4	Oral	14	once-daily	Empagliflozin	410	409	52
(28)	PIONEER 3	NCT02607865	58.0	91.3	32.5	8.6	Oral	3, 7, and 14	once-daily	Sitagliptin	1395	466	78
(26)	PIONEER 4	NCT02863419	56.0	94.0	33.0	7.6	Oral	14	once-daily	Placebo	285	142	57
(23)	SUSTAIN 8	NCT03136484	56.6	90.2	32.3	7.4	Subcutaneous	1	once-weekly	Canagliflozin	392	394	52
(24)	PIONEER 5	NCT02827708	70.0	90.8	32.4	14.0	Oral	14	once-daily	Placebo	163	161	25
(22)	PIONEER 6	NCT02692716	66.0	90.9	32.3	14.9	Oral	14	once-daily	Placebo	1591	1592	87
(25)	PIONEER 7	NCT02849080	57.0	88.6	31.5	8.8	Oral	3, 7, and 14	once-daily	Sitagliptin	253	250	109
(21)	PIONEER 1	NCT02906930	55.0	88.1	31.8	3.5	Oral	3, 7, and 14	once-daily	Placebo	525	178	26
(29)	SUSTAIN 9	NCT03086330	57.0	91.7	31.9	9.7	Subcutaneous	1	once-weekly	Placebo	150	151	30
(30)	PIONEER 8	NCT03021187	61.0	85.9	31.0	15.0	Oral	3, 7, and 14	once-daily	Placebo	547	184	52
(31)	SUSTAIN 10	NCT03191396	59.5	96.9	33.7	9.3	Subcutaneous	1	once-weekly	Liraglutide	290	287	30
(32)	PIONEER 10	NCT03015220	58.0	72.1	26.2	9.4	Oral	3, 7, and 14	once-daily	Dulaglutide	393	65	52
(33)	STEP 2	NCT03552757	55.0	99.8	35.7	8.0	Subcutaneous	1 and 2.4	once-weekly	Placebo	807	403	68
(34)	SURPASS 2	NCT03987919	56.6	93.7	34.2	8.6	Subcutaneous	1	once-weekly	Tirzepatide	469	1409	40
(35)	SUSTAIN CHINA MRCT	NCT03061214	53.1	76.4	27.8	6.4	Subcutaneous	0.5 and 1	once-weekly	Sitagliptin	577	290	30
(36)	–	NCT03951753	61.9	95.0	31.3	12.0	Subcutaneous	1	once-weekly	Placebo	44	28	28
(37)	SUSTAIN 11	NCT03689374	61.2	87.9	31.5	13.4	Subcutaneous	1	once-weekly	Insulin	874	864	52
(38)	SEPRA	NCT03596450	57.4	104.3	35.7	7.4	Subcutaneous	–	once-weekly	Standard of care	634	621	52
(41)	PIONEER 11	NCT04109547	52.0	79.4	23.8	2.5	Oral	3, 7, and 14	once-daily	Placebo	389	131	26
(39)	PIONEER 12	NCT04017832	53.3	79.5	28.6	5.6	Oral	3, 7, and 14	once-daily	Sitagliptin	1082	359	26
(40)	FLOW	NCT03819153	67.0	88.5	31.4	–	Subcutaneous	1	once-weekly	Placebo	1767	1766	177

These studies consisted of subcutaneous administration and oral semaglutide at doses ranging from 0.5 to 14 mg. The average age of the patients ranged from 52 to 70 years, the duration of diabetes ranged from 2.5 to 15 years, and the treatment duration ranged from 26 to 177 weeks. The control group usually received a placebo or a controlled treatment such as insulin, selegiline, or other glucose-lowering therapies. There were 32490 patients with T2D enrolled in total, 18436 in the semaglutide group and 14054 in the control group.

### 3.2 Arrhythmic outcomes

**Figure 2:**
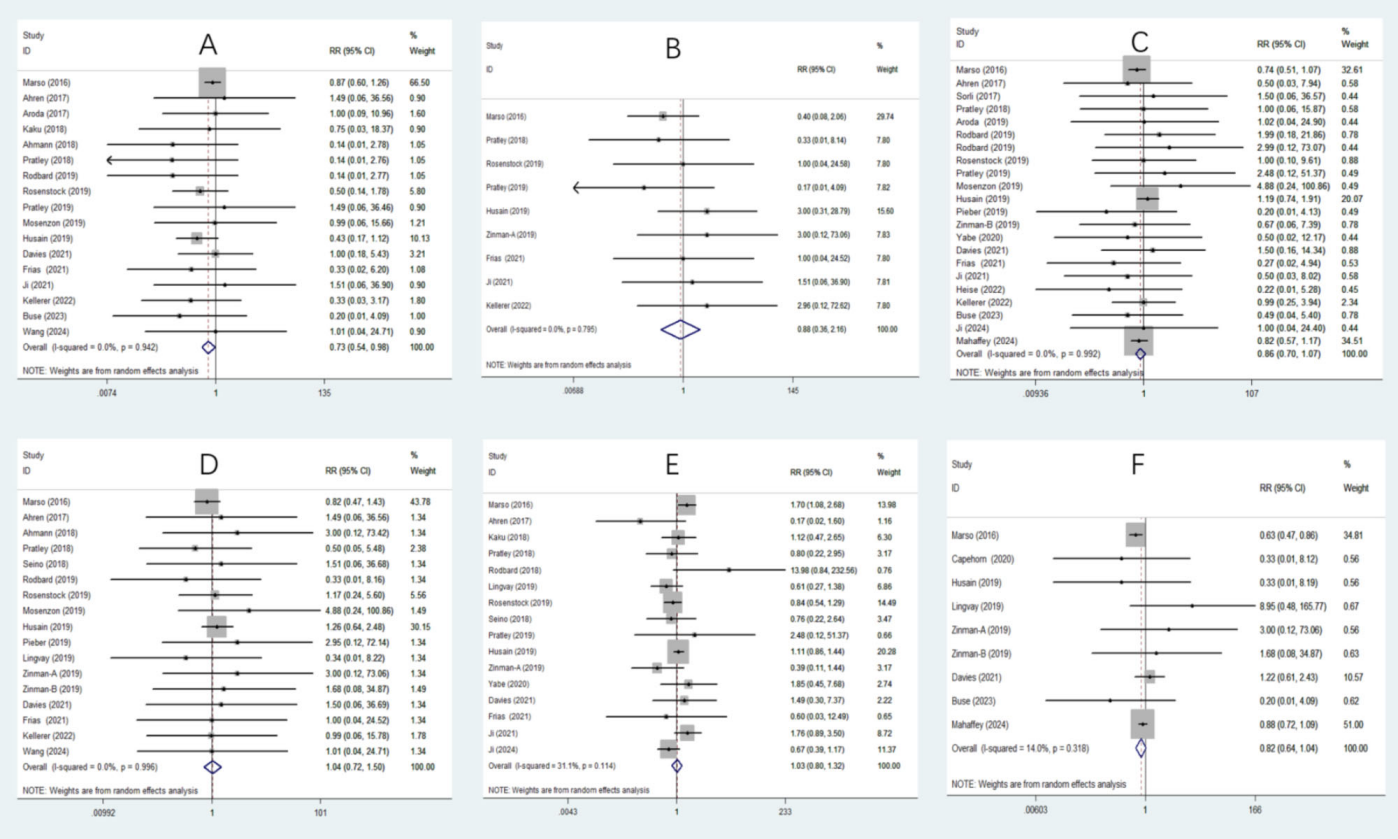
Forest plot depicting the risk of various outcomes associated with semaglutide treatment compared with the control. **(A)** Atrial fibrillation (AF): Semaglutide significantly reduces the risk of AF (RR 0.73, 95% CI 0.54 to 0.98), **(B)** Atrial flutter (AFL): No significant effect was observed (RR 0.88, 95% CI 0.36 to 2.16), **(C)** Nonfatal myocardial infarction: No significant effect was observed (RR 0.86, 95% CI 0.70 to 1.07), **(D)** Unstable angina pectoris: No significant effect was observed (RR 1.04, 95% CI 0.72 to 1.50), **(E)** Retinopathy complications: No significant effect was observed (RR 1.03, 95% CI 0.80 to 1.32), **(F)** New or worsening nephropathy: No significant effect was observed (RR 0.82, 95% CI 0.64 to 1.04).

In the meta-analysis examining arrhythmic outcomes, semaglutide significantly reduced the risk of AF (RR 0.73, 95% CI 0.54 to 0.98) (**Figure 2A**). However, no significant effects were found for AFL (RR 0.88, 95% CI 0.36 to 2.16) (**Figure 2B**), ventricular tachycardia (RR 1.77, 95% CI 0.71 to 4.39) (**Supplementary Figure 1A**), supraventricular tachycardia (RR 1.22, 95% CI 0.41 to 3.64) (**Supplementary Figure 1B**), ventricular fibrillation (RR 0.95, 95% CI 0.20 to 4.55) (**Supplementary Figure 1C**), or sinus node dysfunction (RR 0.74, 95% CI 0.14 to 3.94) (**Supplementary Figure 1D**). Regarding AV blocks, semaglutide significantly reduced complete AV block (RR 0.22, 95% CI 0.06 to 0.80) (**Supplementary Figure 1E**) but had no significant effect on first-degree (RR 1.60, 95% CI 0.20 to 13.01) (**Supplementary Figure 2F**) or second-degree AV block (RR 0.64, 95% CI 0.23 to 1.80) (**Supplementary Figure 1G**).

### 3.3 Major CV outcomes

In the meta-analysis assessing major CV outcomes, semaglutide significantly reduced the risk of death from CV causes (RR 0.76, 95% CI 0.58 to 0.98) (**Supplementary Figure 2A**) and revascularization (RR 0.68, 95% CI 0.52 to 0.88) (**Supplementary Figure 2B**). However, there were no significant effects on nonfatal myocardial infarction (RR 0.86, 95% CI 0.70 to 1.07) (**Figure 2C**), nonfatal stroke (RR 0.83, 95% CI 0.52 to 1.33) (**Supplementary Figure 2C**), unstable angina pectoris (RR 1.04, 95% CI 0.72 to 1.50) (**Figure 2D**), or HF (RR 1.01, 95% CI 0.76 to 1.35) (**Supplementary Figure 2D**).

### 3.4 Microvascular outcomes

In terms of microvascular outcomes, semaglutide did not significantly affect retinopathy complications (RR 1.03, 95% CI 0.80 to 1.32) (**Figure 2E**). However, it tended to reduce the risk of new or worsening nephropathy (RR 0.82, 95% CI 0.64 to 1.04) (**Figure 2F**), although this result was also not statistically significant. Despite the lack of statistical significance, this observed reduction reflects a favorable trend toward improved microvascular outcomes with semaglutide and is clinically relevant, as the prevention of microvascular outcomes is a critical concern in the management of T2D. Therefore, further research focusing on nephropathy outcomes will be essential to confirm and elaborate on these promising findings.

### 3.5 Subgroup analysis

We performed subgroup analyses based on different administration types, treatment comparisons, and treatment durations. The RR values for each subgroup and the degree of heterogeneity are shown in **Table 2**.

**Table 2:** Subgroup analyses based on different administration types, treatment comparisons, and treatment durations.

Subgroup	No. of studies	RR (95% CI)	Heterogeneity (*I* ^2^%)	*p*
Atrial fibrillation				
Oral	6	0.49 (0.25, 0.97)	0	0.894
Subcutaneous	11	0.80 (0.57, 1.12)	0	0.892
semaglutide vs placebo	6	0.81 (0.58, 1.13)	0	0.842
semaglutide vs other drug	11	0.43 (0.21, 0.90)	0	0.955
Duration < 52 weeks	6	0.65 (0.20, 2.10)	0	0.873
Duration ≥ 52 weeks	11	0.73 (0.53, 1.00)	0	0.781
Atrial flutter				
Oral	3	1.09 (0.21, 5.61)	4.3	0.352
Subcutaneous	6	0.79 (0.27, 2.34)	0	0.785
semaglutide vs placebo	4	0.81 (0.22, 2.95)	16	0.312
semaglutide vs other drug	5	1.08 (0.25, 4.52)	0	0.917
Duration < 52 weeks	4	1.10 (0.22, 5.48)	0	0.811
Duration ≥ 52 weeks	5	0.79 (0.27, 2.34)	0	0.466
Ventricular tachycardia				
Oral	3	1.00 (0.15, 6.35)	0	0.637
Subcutaneous	3	2.10 (0.68, 6.50)	4.6	0.351
semaglutide vs placebo	3	1.68 (0.59, 4.77)	0	0.519
semaglutide vs other drug	3	2.06 (0.30, 13.94)	6.3	0.344
Duration < 52 weeks	1	8.98 (0.36, 220.08)	Not estimable	Not estimable
Duration ≥ 52 weeks	5	1.53 (0.59, 3.95)	0	0.66
Supraventricular tachycardia				
Oral	2	0.57 (0.06, 5.55)	0	0.633
Subcutaneous	3	1.53 (0.44, 5.33)	0	0.906
semaglutide vs placebo	3	1.10 (0.31, 3.82)	0	0.728
semaglutide vs other drug	2	1.22 (0.41, 3.64)	0	0.638
Duration < 52 weeks	0	Not estimable	Not estimable	Not estimable
Duration ≥ 52 weeks	5	0.79 (0.25, 0.99)	0	0.914
Second degree AV block				
Oral	4	0.66 (0.11, 3.87)	31.7	0.608
Subcutaneous	3	0.53 (0.12, 2.36)	0	0.222
semaglutide vs placebo	3	1.58 (0.41, 6.06)	0	0.771
semaglutide vs other drug	4	0.17 (0.03, 0.88)	0	0.85
Duration < 52 weeks	0	Not estimable	Not estimable	Not estimable
Duration ≥ 52 weeks	7	0.64 (0.23, 1.79)	0	0.483
Complete AV block				
Oral	1	0.33 (0.01, 8.18)	Not estimable	Not estimable
Subcutaneous	4	0.20 (0.05, 0.83)	0	0.924
semaglutide vs placebo	2	0.23 (0.04, 1.39)	0	0.795
semaglutide vs other drug	3	0.21 (0.03, 1.33)	0	0.788
Duration < 52 weeks	1	0.33 (0.01, 8.13)	Not estimable	Not estimable
Duration ≥ 52 weeks	4	0.20 (0.05, 0.83)	0	0.924
Nonfatal myocardial infarction				
Oral	11	1.18 (0.78, 1.79)	0	0.969
Subcutaneous	11	0.77 (0.60, 0.99)	0	0.995
semaglutide vs placebo	10	0.87 (0.69, 1.08)	0	0.806
semaglutide vs other drug	12	0.78 (0.38, 1.59)	0	0.988
Duration < 52 weeks	8	0.81 (0.28, 2.36)	0	0.899
Duration ≥ 52 weeks	14	0.86 (0.69, 1.07)	0	0.956
Nonfatal stroke				
Oral	1	0.75 (0.35, 1.58)	Not estimable	Not estimable
Subcutaneous	3	0.83 (0.43, 1.60)	67.4	0.046
semaglutide vs placebo	3	0.86 (0.53, 1.38)	62.5	0.069
semaglutide vs other drug	1	0.16 (0.01, 4.07)	Not estimable	Not estimable
Duration < 52 weeks	0	Not estimable	Not estimable	Not estimable
Duration ≥ 52 weeks	4	0.83 (0.51, 1.33)	53.4	0.092
Unstable angina pectoris				
Oral	7	1.29 (0.73, 2.28)	0	0.94
Subcutaneous	10	0.87 (0.53, 1.43)	0	0.99
semaglutide vs placebo	7	1.04 (0.69, 1.57)	0	0.856
semaglutide vs other drug	10	1.01 (0.43, 2.36)	0	0.986
Duration < 52 weeks	6	1.34 (0.39, 4.55)	0	0.892
Duration ≥ 52 weeks	11	1.01 (0.68, 1.49)	0	0.981
Heart failure				
Oral	4	0.92 (0.53, 1.59)	0	0.934
Subcutaneous	9	1.04 (0.74, 1.47)	0	0.884
semaglutide vs placebo	5	0.99 (0.73, 1.34)	0	0.593
semaglutide vs other drug	8	1.23 (0.44, 3.45)	0	0.988
Duration < 52 weeks	4	0.66 (0.14, 3.08)	0	0.822
Duration ≥ 52 weeks	9	1.02 (0.76, 1.38)	0	0.931
Retinopathy complications				
Oral	5	0.97 (0.77, 1.22)	6.8	0.368
Subcutaneous	11	1.05 (0.69, 1.60)	37.9	0.097
semaglutide vs placebo	6	1.28 (0.81, 2.03)	43	0.118
semaglutide vs other drug	10	0.87 (0.67, 1.14)	2.3	0.418
Duration < 52 weeks	7	0.92 (0.51, 1.65)	43.5	0.101
Duration ≥ 52 weeks	9	1.08 (0.83, 1.41)	25.4	0.218
New or worsening nephropathy				
Oral	2	0.78 (0.08, 7.06)	0	0.472
Subcutaneous	7	0.83 (0.61, 1.12)	31.7	0.186
semaglutide vs placebo	6	0.81 (0.66, 1.00)	10.1	0.351
semaglutide vs other drug	3	0.87 (0.07, 9.97)	47.6	0.149
Duration < 52 weeks	2	1.00 (0.10, 9.56)	0	0.34
Duration ≥ 52 weeks	7	0.82 (0.61, 1.09)	28.2	0.213

AV, atrioventricular.

Subgroup analysis revealed that oral semaglutide significantly reduced the risk of AF (RR 0.49, 95% CI 0.25 to 0.97), whereas subcutaneous semaglutide did not have a significant effect (RR 0.80, 95% CI 0.57 to 1.12). Compared with other drugs, semaglutide also significantly reduced the risk of AF (RR 0.43, 95% CI 0.21 to 0.90). For nonfatal myocardial infarction, subcutaneous semaglutide significantly reduced the risk (RR 0.77, 95% CI 0.60 to 0.99), whereas oral semaglutide had no significant effect (RR 1.18, 95% CI 0.78 to 1.79). Additionally, subcutaneous administration significantly lowered the risk of complete AV block (RR 0.20, 95% CI 0.05 to 0.83). In terms of retinopathy complications, neither oral nor subcutaneous semaglutide had a significant impact, although studies on subcutaneous semaglutide demonstrated moderate heterogeneity (*I*² = 37.9%). For new or worsening nephropathy, subcutaneous semaglutide did not significantly reduce the risk (RR 0.83, 95% CI 0.61 to 1.12), but it had a borderline significance compared with placebo (RR 0.81, 95% CI 0.66 to 1.00). For outcomes such as HF and unstable angina pectoris, neither administration type nor control comparisons showed significant risk reduction. Overall, oral semaglutide demonstrated notable benefits in patients with AF, whereas subcutaneous semaglutide was particularly effective in reducing the risks of nonfatal myocardial infarction and complete AV block. Most analyses revealed no heterogeneity (*I*² = 0), suggesting high consistency, but outcomes with high heterogeneity, such as retinopathy and nephropathy, should be interpreted cautiously.

### 3.6 Publication bias and meta-regression

Funnel plots were generated for the outcomes of AF, nonfatal myocardial infarction, unstable angina pectoris, HF, and retinopathy complications, and they were found to be largely symmetrical (**Supplementary Figure 3**). Furthermore, the results of the Egger test yielded p values of 0.373, 0.105, 0.503, 0.509, and 0.561, respectively. These findings suggest the absence of publication bias for these outcomes.

**Figure 3:**
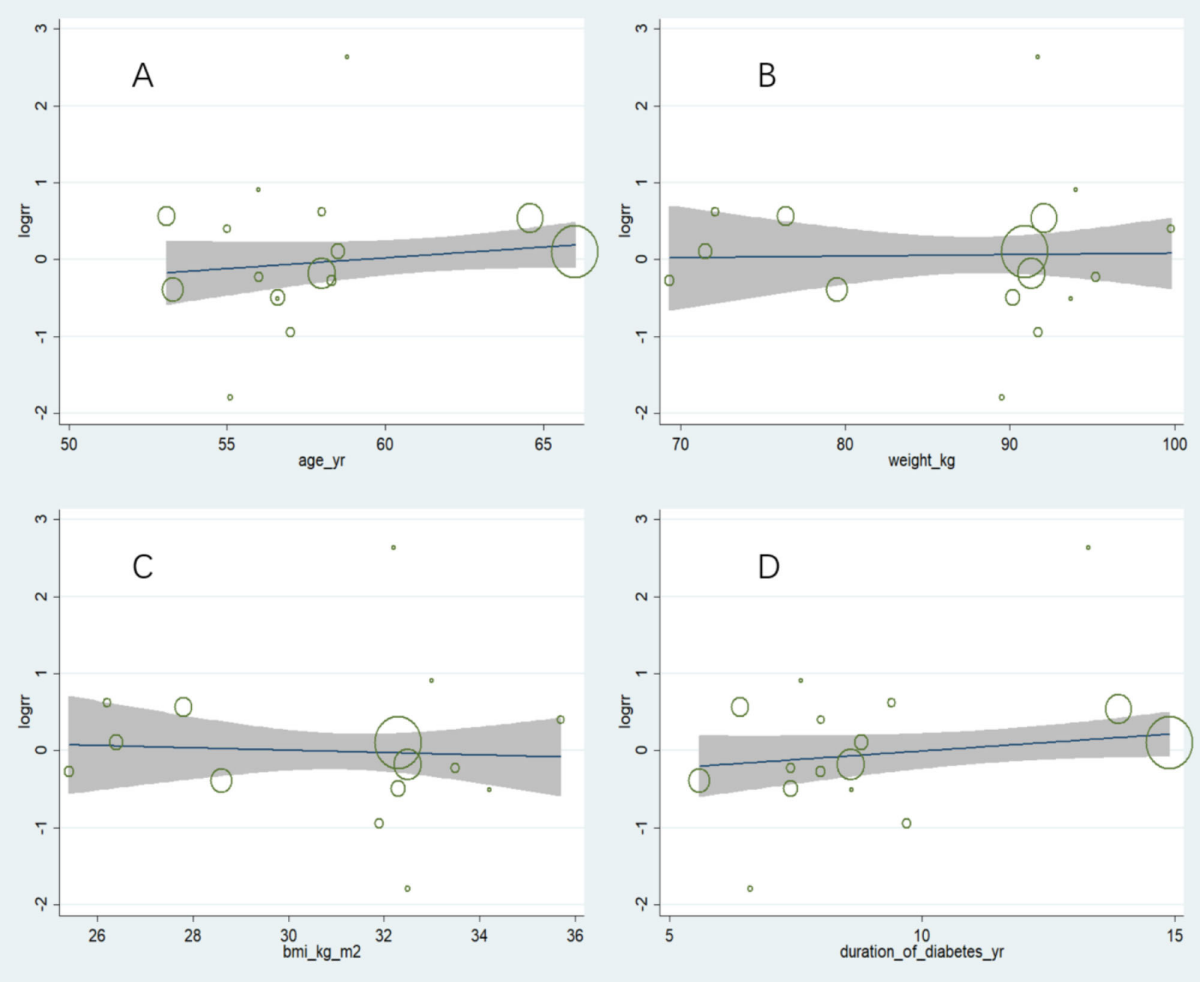
Meta-regression analysis of factors potentially associated with heterogeneity in retinopathy complications. **(A)** Age: No significant correlation observed (p = 0.244), **(B)** Weight: No significant correlation observed (p = 0.873), **(C)** BMI: No significant correlation observed (p = 0.576), **(D)** Duration of diabetes: No significant correlation observed (p = 0.133). Overall, these results indicate that baseline characteristics did not account for the observed heterogeneity in retinopathy outcomes.

Owing to the observed heterogeneity in the outcome of retinopathy complications, a meta-regression was conducted considering baseline factors such as age, weight, BMI, and duration of diabetes (**Figure 3**). The results indicated that heterogeneity was not related to these baseline factors, with p values of 0.244, 0.873, 0.576, and 0.133, respectively.

### 3.7 Assessment of study quality

The assessment of study quality, as summarized in the risk of bias summary (**Supplementary Figure 4**), indicates that most included studies were rated as having a low risk of bias across key domains such as random sequence generation, allocation concealment, and blinding. This suggests that the overall methodological rigor of the studies was satisfactory. However, a few studies presented a high risk of bias, particularly in the domains of blinding and incomplete outcome data, which could introduce potential limitations. Additionally, some studies were rated as having an unclear risk of bias due to insufficient reporting of methodological details.

## 4 Discussion

This systematic review and meta-analysis provides an updated and detailed evaluation of the effects of semaglutide on arrhythmic, major CV, and microvascular outcomes in patients with T2D. These findings confirm the strong potential of semaglutide as a therapeutic agent that extends beyond glycemic control to exert pleiotropic benefits, particularly in reducing the risk of arrhythmic and major CV outcomes. These results align with the growing body of evidence suggesting that GLP-1 receptor agonists hold promise for mitigating CV outcomes in patients with T2D.

The observed reduction in AF risk with semaglutide, particularly with oral administration, underscores its potential role in mitigating arrhythmic outcomes in patients with T2D. However, no significant effects were found for other arrhythmic outcomes, such as ventricular tachycardia and AV blocks, suggesting a need for further studies focusing on these less common outcomes. In terms of major CV outcomes, semaglutide demonstrated significant reductions in death from CV causes and revascularization. These findings reinforce its role as an essential therapy for CV risk reduction in patients with T2D. However, the lack of significant effects on nonfatal myocardial infarction, nonfatal stroke, and HF highlights the complexity of CV disease in diabetes and suggests that the benefits of semaglutide may be more pronounced for certain outcomes than others. Subgroup analyses revealed notable differences on the basis of administration types and treatment comparisons, emphasizing the need for personalized approaches to semaglutide therapy. For microvascular outcomes, semaglutide had no significant effect on retinopathy complications or new or worsening nephropathy. The findings indicate semaglutide’s benefits are primarily CV rather than microvascular. In interpreting our results, it is critical to address the potential impact of heterogeneity in retinopathy outcomes among the included studies. The variability in these outcomes may influence the conclusions drawn from our meta-analysis. Several factors could contribute to this heterogeneity, including differences in study populations, methods of assessing retinopathy, and treatment durations. For example, the populations studied may vary significantly in terms of demographic characteristics, which could influence the prevalence and severity of retinopathy. Additionally, variations in the methodologies utilized to evaluate retinopathy—ranging from clinical assessments to imaging techniques—can lead to inconsistencies in the outcomes reported. Furthermore, differences in treatment duration and patient adherence to semaglutide may introduce further variability in the results. Acknowledging this complexity underscores the need for caution when generalizing our findings and emphasizes the importance of standardized approaches in future research.

The observed CV and arrhythmic benefits of semaglutide in patients with T2D are likely mediated through multiple interrelated mechanisms. First, the ability of semaglutide to improve glycemic control and reduce body weight contributes to a favorable metabolic environment that reduces inflammation, oxidative stress, and insulin resistance—key drivers of CV and arrhythmic outcomes (42). Second, semaglutide has been shown to improve endothelial function and reduce arterial stiffness (43), which may attenuate the progression of atherosclerosis and lower the risk of adverse CV outcomes. This vascular benefit could also play a role in reducing atrial remodeling, a critical factor in the development of AF. Third, the antihypertensive effects of semaglutide (44), achieved through reductions in blood pressure, may alleviate hemodynamic stress on the heart, particularly in the atrial and ventricular chambers. By mitigating chronic pressure overload, semaglutide may help prevent arrhythmogenic structural changes such as atrial dilation and fibrosis. Fourth, emerging evidence suggests that GLP-1 receptor agonists, including semaglutide, may have direct antiarrhythmic effects (45). These could involve the modulation of ion channel activity, the suppression of sympathetic nervous system overactivation, or reductions in ectopic electrical activity. These direct effects could explain the significant reduction in AF and complete AV block observed in this analysis. Finally, the anti-inflammatory and antifibrotic effects of semaglutide may contribute to cardiac structural remodeling (46), reducing the likelihood of arrhythmic and CV outcomes. Chronic inflammation and fibrosis are well-established contributors to both major CV and microvascular outcomes in patients with T2D, and the ability of semaglutide to dampen these processes likely plays a central role in its cardioprotective profile. Although these mechanisms are plausible and supported by preclinical and early clinical studies, further research is necessary to clarify the precise pathways through which semaglutide exerts its CV and antiarrhythmic benefits.

The differential effects observed with oral semaglutide compared with the subcutaneous formulation may be attributed to several factors. One potential explanation is the difference in pharmacokinetics between the two routes of administration. Oral semaglutide is absorbed in the gastrointestinal tract, which may lead to distinct circulating concentrations and metabolic effects that are more effective in preventing arrhythmias such as AF. In contrast, subcutaneous semaglutide delivers a steady release into the bloodstream, which may afford better protection against acute CV outcomes such as myocardial infarction and complete heart block. Furthermore, these differences in administration methods could influence patient compliance and medication adherence, further impacting clinical outcomes. Therefore, understanding the underlying mechanisms of these varied effects may inform clinical practice and optimize treatment strategies for patients with T2D.

This study builds on previous work to further understand the role of semaglutide in several CV and metabolic domains. Previous meta-analyses have focused on the role of semaglutide in the prevention of AF. For example, Zhang et al. (47) reported that, compared with placebo, semaglutide reduced the incidence of AF by 30% in patients with T2D, obesity, or overweight (RR 0.70,95% CI 0.52 to 0.95), emphasizing its effectiveness in mitigating the risk of arrhythmia, which is similar to our results. In contrast, de Oliveira Almeida et al. (48) reported that semaglutide did not reduce the incidence of AF in patients with obese or overweight compared with placebo (RR 0.49, 95% CI 0.17 to 1.43), which may indicate that the main effect in such patients is diabetes control rather than improvement in CV outcomes. Furthermore, Saglietto et al. (8) reported that semaglutide reduced the risk of AF in patients at high CV risk with subcutaneous administration (RR 0.59, 95% CI 0.39 to 0.91) but not with oral administration (RR 0.53, 95% CI 0.23 to 1.24), which contradicts our results. On the basis of the uniqueness of these studies, our study expands the underexplored area: the specific effect of semaglutide on various arrhythmic outcomes, such as AFL, conduction block and ventricular arrhythmias. Our findings confirm the broad role of semaglutide in arrhythmia prevention, while revealing subtle differences in its efficacy depending on the route of administration. Moreover, major CV and microvascular outcomes have been of greater interest. A study by Sattar et al. (49) revealed that GLP-1 agonists significantly reduced the risk of HF in patients with T2D (RR 0.89, 95% CI 0.82 to 0.98), whereas Qin and Song (50) reported that they reduced the risk of nonfatal stroke (RR 0.85; 95% CI 0.77 to 0.94). However, the results of these two studies contrast with our study, possibly because they included only two studies and one study of semaglutide, whereas other GLP-1 receptor agonists may have different effects on these outcomes. Therefore, it is particularly important to analyze each GLP-1 receptor agonist in more detail, especially with respect to its effect on adverse outcomes. In addition, Wang et al. (51) reported that semaglutide did not affect the risk of retinopathy complications in patients with T2D (RR 1.14, 95% CI 0.98 to 1.33) but provided new insights into its protective effect on the microvasculature. Although our meta-analysis did not reach statistical significance for this outcome either, the subgroup trend we observed suggests that semaglutide may reduce the progression of other microvascular outcomes (new or worsening nephropathy), especially in long-term treatment. Furthermore, recent studies (52, 53) have demonstrated long-term benefits of semaglutide on CV outcomes, reinforcing the concept that GLP-1 receptor agonists may reduce the burden of CV outcomes in patients with T2D beyond mere glycemic control. Overall, this study not only confirms the CV benefits of semaglutide but also emphasizes its potential to reduce the risk of arrhythmias. These findings fill an important gap in the literature and pave the way for further research.

This meta-analysis has several strengths. First, one of the key strengths of our meta-analysis was the comprehensive assessment of the effects of semaglutide on a broad spectrum of outcomes, including arrhythmic, major CV, and microvascular outcomes. Unlike previous analyses, we placed particular emphasis on arrhythmic outcomes, providing valuable insights into the potential benefits of semaglutide in managing these outcomes, which have not been extensively explored before. Second, our study included both oral and subcutaneous semaglutide formulations, which allows for a more detailed comparison of their effects. This adds depth to our analysis, as it helps differentiate the potential outcomes on the basis of administration type, which previous meta-analyses have not adequately taken into account. Third, our study draws on the most recent and relevant RCTs, ensuring that our findings are reflective of the latest evidence available. This strengthens the relevance and applicability of our results in real-world clinical settings. However, our analysis also has several limitations. First, some outcomes, particularly microvascular outcomes such as retinopathy complications, showed heterogeneity. While we conducted meta-regression to explore potential sources of this heterogeneity, further studies are needed to confirm and understand this heterogeneity fully. Second, the variability in study designs, including differences in treatment durations, control interventions, and patient populations, could limit the generalizability of our findings. These differences may introduce biases that affect how applicable our conclusions are to broader clinical practice. Finally, while the majority of studies had a low risk of bias, a few studies showed high risk, particularly in the areas of blinding and incomplete outcome data. These issues could influence the robustness of the results and should be considered when the findings are interpreted. In conclusion, while our meta-analysis provides valuable insights into the effects of semaglutide, particularly for arrhythmic outcomes, the limitations outlined above must be taken into account.

Future research should focus on longer-term studies to evaluate the sustained effects of semaglutide on CV and microvascular outcomes, as well as to explore its full range of benefits and risks, considering the chronic nature of T2D and its associated complications. In particular, large clinical trials that specifically target AF or other arrhythmias as primary endpoints will be necessary to improve our understanding of the antiarrhythmic benefits of semaglutide. Additionally, mechanistic studies are needed to clarify the underlying pathways through which semaglutide exerts its antiarrhythmic and cardioprotective effects, potentially identifying new therapeutic targets. Subgroup analyses examining diverse populations, including those with advanced diabetes or preexisting CV disease, will also be essential in personalizing treatment and optimizing outcomes for patients with varying needs and comorbidities.

## 5 Conclusions

This systematic review and meta-analysis demonstrated that semaglutide significantly reduces the risk of AF, complete AV block, death from CV causes, and revascularization in patients with T2D. These findings highlight its cardioprotective potential beyond glycemic control. While no significant effects were observed for other arrhythmic or microvascular outcomes, the overall evidence supports semaglutide as a valuable therapeutic option for managing CV risk in patients with T2D. Further research is needed to explore its long-term impact and address heterogeneity in specific outcomes.

## Data Availability

The raw data supporting the conclusions of this article will be made available by the authors, without undue reservation.

## Supplementary material

Supplementary Material S1 Search strategies.

Supplementary Material S2 Forest plot of arrhythmic outcomes.

Supplementary Material S3 Forest plot of major cardiovascular outcomes.

Supplementary Material S4 Funnel plot.

Supplementary Material S5 The risk of bias summary.

Supplementary Material S6 PRISMA checklist.
